# Attitude, perception, willingness, motivation and barriers to practice-based research: A cross-sectional survey of hospital pharmacists in Lahore, Punjab, Pakistan

**DOI:** 10.1371/journal.pone.0203568

**Published:** 2018-09-07

**Authors:** Muhammad Rehan Sarwar, Anum Saqib, Tayyab Riaz, Haleema Aziz, Mosab Arafat, Hamna Nouman

**Affiliations:** 1 Akhtar Saeed College of Pharmaceutical Sciences, Lahore, Pakistan; 2 Department of Pharmacy, The Islamia University of Bahawalpur, Bahawalpur, Punjab, Pakistan; 3 College of Pharmacy, Al Ain University of Science and Technology, Al Ain, Abu Dhabi, United Arab Emirates; Charles Sturt University, AUSTRALIA

## Abstract

**Background and objectives:**

Practice-based research (PBR) is of pivotal importance for hospital pharmacists which not only up-grades the profession but also improves the patient care. This study aimed to evaluate the attitude, perception, willingness, motivation and barriers to PBR among hospital pharmacists in Pakistan.

**Methods:**

A descriptive, cross sectional study design was employed. Data were collected between 1^st^ December, 2017 and 1^st^ March, 2018 from 130 hospital pharmacists employed in 41 hospitals of Lahore, Pakistan. A survey instrument comprising of six sections was designed to determine the attitude, perception, willingness, motivation and barriers to PBR. Data were analyzed by using Statistical Package for Social Sciences (IBM SPSS Statistics for Windows, version 21.0, Armonk, NY: IBM Corp.). The normality of the data was determined through Shapiro-Wilks and Kolmogorov-Smirnov tests. Independent Samples Mann-Whitney U Test and Independent Samples Kruskal-Wallis Test were carried out to test if there were differences among the characteristics of the hospital pharmacists. Logistic regression analysis was used to figure out the factors associated with attitude, perceptions, willingness and motivation towards PBR. A *p-value* <0.05 was used for statistical significance of differences.

**Results:**

A total of 141 pharmacists were approached. Among them, 130 responded to the survey (response rate 92%). Out of a maximum score i.e., 5 (100%) the respondents obtained a median score of 4 (IQR = 0) for attitude, perception and motivation towards PBR; whereas, a median score of 4 (IQR = 1) was obtained for willingness thus demonstrating fair positive attitude, good perceptions, increased motivation and willingness towards PBR. The most common barrier limiting the pharmacists’ participation in PBR was lack of time (23.8%) followed by lack of incentives (16.2%) and lack of support (14.6%). Results of the logistic regression analysis revealed that hospital pharmacists practicing in the inpatient settings had 4.56 times more positive attitude towards PBR (OR = 4.56, 95%CI = 1.07─19.42, *p-value* = 0.040) as compared to those practicing in the outpatient settings. The male hospital pharmacists (OR = 8.86, 95%CI = 1.15–53.74, *p-value* = 0.017), those practicing in the outpatient (OR = 23.51, 95%CI = 2.04─271.53, *p-value* = 0.011) and inpatient settings had increased motivation towards PBR (OR = 12.24, 95%CI = 1.61─94.66, *p-value* = 0.016).

**Conclusion:**

Despite the presence of several barriers, the respondents had fair positive attitude, good perceptions, increased motivation and willingness towards PBR which is a promising finding.

## Introduction

Medical research demands continuous up-gradation of professional practice and knowledge [1]. Thus, it is very crucial to develop the interest of pharmacists towards practice-based research (PBR). Pharmacy practice research is defined as, “a component of health services research that focuses on the assessment and evaluation of pharmacy practice” [2]. PBR not only improves the quality of pharmaceutical care but also helps to extend its scope [3]. In the past, pharmacists were reluctant to conduct research [4] but this trend has been changed with the passage of time. PBR is now being increasingly acknowledged and supported by pharmacy organizations globally [5]. The American College of Clinical Pharmacy (ACCP) is working to pave ways for the betterment of human health and quality of life by encouraging pharmacists to be a part of the research [6]. Similarly, the Canadian Society of Hospital Pharmacists has also developed a research and education foundation for enhancing the research skills of pharmacists [3]. It is essential to conduct more research for spreading awareness about the concept of PBR and its impact on the betterment of the community. Most of the pharmacists practicing in developing countries lack interest in research activities. In contrast to this a Qatari study showed that more participants were ardent to participate in PBR, though some obstacles were also there [7].

The social cognitive theory states that researchers are greatly influenced by the environmental and personal factors [8]. Environmental factors are related to the financial rewards, support and encouragement from concerned authorities. The personal factors are the one’s own interests and curiosity, desire to flourish and to improve the skills and knowledge related to disease management. Both of these factors play a pivotal role in the researchers work. According to this theory the thoughts, beliefs and feelings of a person has a strong influence on his behavior. If a person has positive attitude towards research, understands the importance of research in uplifting the profession and is cognizant of the motivators and barriers in research then he could play a pivotal role in conducting PBR. Literature suggests that the involvement of pharmacists in PBR can lead to better patient outcomes; however, the pharmacists have to overcome certain barriers in this regard. These barriers include inadequate training, lack of skills and competency required for PBR [9–11]. Although, number of hospital pharmacists is continuously increasing with each passing year but there is scarcity of data that reflects the ongoing scenario of PBR in Pakistan or other developing countries. Most of the literature pertaining to PBR has been reported from developed countries [12, 13]. In such circumstances, it is necessary to scrutinize the opinions and inclination of hospital pharmacists in Pakistan about their participation in PBR. This aspect has not yet been explored in Pakistan and warrants attention. Therefore, the focus of this study is to evaluate the attitude, perception, willingness, motivation and barriers to PBR among hospital pharmacists in Pakistan.

## Methods

### Study design and settings

A descriptive, cross sectional study design was employed. Out of the total 66 hospitals present in Lahore, hospital pharmacists are employed in 41 hospitals (23 private, 17 public, and 1 military hospital). Lahore is the 32^nd^ most populated city in the world and second most populated city in Pakistan, with the population of 15,245,000 [14]. This city has a total of 48 private, 17 public, and 1 military hospital for catering the needs of the growing population [15]. The study was conducted among those 41 hospitals of Lahore, Pakistan where hospital pharmacists were employed.

### Study population and sample size

Study population consisted of hospital pharmacists employed in private and government hospitals of Lahore. The total sampling strategy was used for the current study. All the hospital pharmacists (n = 141) working in Lahore were approached out of which 130 hospital pharmacists consented to participate in this study.

### Data collection

Data were collected between 1^st^ December, 2017 and 1^st^ March, 2018. An additional note was attached with each questionnaire which defined the aim of the research. An extensive literature review of previously published findings was executed [13, 16, 17] and a survey instrument was designed [S1 Appendix]. Three experienced pharmacy practitioners and professors assessed the survey instrument for face and content validity. Centered on their opinions, the questionnaire was adjusted and piloted on 10 hospital pharmacists’ prior to its administration among the study participants.

The survey instrument had six distinct sections. The first two sections sought the attitude, perception and willingness of hospital pharmacists towards PBR and were measured on a five point Likert scale (5 = strongly agree, 4 = agree, 3 = neutral, 2 = disagree and 1 = strongly disagree). The third section included the factors that motivated the pharmacist to be a part of research and was also measured by the Likert scale. The fourth section examined the barriers in executing research such as lack of time, support and knowledge etc. The fifth section explored the main areas of interest for research and the final section comprised of the participant’s demographics.

### Data analysis

Data were analyzed by using Statistical Package for Social Sciences (IBM SPSS Statistics for Windows, version 21.0, Armonk, NY: IBM Corp.). Internal consistency of the questionnaire was measured by Cronbach’s alpha, while reproducibility was evaluated using intra-class correlation for each item in the attitude, perceptions, willingness, and motivation scales, with acceptable values ≥0.6. Calculation for Cronbach’s alpha was made as 0.77 for attitude, 0.73 for perceptions, 0.75 for willingness, and 0.73 for motivation section. Descriptive statistics such as frequencies, percentages, median and interquartile ranges were used to analyze the data. Moreover, the normality of the data was determined through Shapiro-Wilks and Kolmogorov-Smirnov tests. Outcomes regarding attitude, perceptions, willingness and motivation towards PBR were dichotomized as “Positive” versus “Negative”, “Good” versus “Poor”, “More” versus “Less”, and “High” versus “Low”, respectively. Median scores of ≥3 were considered as “Positive”, “Good”, “More”, and “High”; whereas, scores <3 were considered as “Negative”, “Poor”, “Less”, and “Low”, respectively. Independent Samples Mann-Whitney U Test and Independent Samples Kruskal-Wallis Test were carried out to test if there were differences among characteristics of the hospital pharmacists with regard to their attitudes, perceptions, willingness and motivation towards PBR. Logistic regression analysis was performed to figure out the factors associated with attitude, perceptions, willingness and motivation towards PBR. Results were expressed as Odds Ratio (OR) accompanied by 95% Confidence Intervals (95%CI) and a p-value <0.05 was used for statistical significance of differences.

### Ethics approval and consent to participate

The ethical approval was obtained from the Pharmacy Research Ethics Committee (PREC) at the Akhtar Saeed College of Pharmaceutical Sciences (Reference: 16-2017/PREC, dated November 23, 2017). Before initiating the study, the purpose and protocols were thoroughly explained to participants and written consents were also obtained.

## Results

### Characteristics of the participants

A total of 141 hospital pharmacists were approached. Out of them, 130 participants agreed to participate in the survey (response rate = 92%). Most of the respondents were male (n = 75, 57.7%) and had a professional experience of less than 2 years (n = 67, 51.5%). Most of the respondents had neither any clinical training (n = 74, 56.9%) nor other board certifications (n = 108, 83.1%). Majority of them were interested in surgical unit (n = 23, 17.7%), employed in inpatient departments (n = 66, 50.8%), and had major research interest in the field of therapeutics (n = 24, 18.5%) (Table 1).

**Table 1:** Characteristics of the participants.

Variables		N (%)
**Age**	less than 25	67 (51.5)
	25–30	40 (30.8)
	31–35	12 (9.2)
	36–40	5 (3.8)
	41–45	4 (3.1)
	46–50	2 (1.5)
**Gender**	Male	75 (57.7)
	Female	55 (42.3)
**Qualification**	Pharm.D*	108 (83.1)
	M.Phil^†^	17 (13.1)
	PhD^‡^	5 (3.8)
**Years of experience in pharmacy**	< 2	67 (51.5)
	2–5	42 (32.3)
	6–10	14 (10.8)
	>10	7 (5.4)
**Clinical training**	Yes	56 (43.1)
	No	74 (56.9)
**Other board certified qualifications**	Yes	22 (16.9)
	No	108 (83.1)
**Current area of practice in hospital**	Outpatient	32 (24.6)
	Inpatient	66 (50.8)
	Emergency	17 (13.1)
	Others	15 (11.5)
**Area of interest**	Pediatrics/neonates	10 (7.7)
	ICU^§^	8 (6.2)
	Cardiac	18 (13.8)
	Transplant	7 (5.4)
	Nephrology	9 (6.9)
	Surgery	23 (17.7)
	Internal medicine	20 (15.4)
	Geriatrics	12 (9.2)
	Infectious diseases	12 (9.2)
	Oncology	11 (8.5)
**Research interest**	Pharmacy Administration	14 (10.8)
	Basic Science (Pharmacogenomics, New structural drugs)	15 (11.5)
	Pharmacoeconomics/Epidemiology	13 (10.0)
	Pharmacy practice	22 (16.9)
	Hospital Pharmacy	22 (16.9)
	Therapeutics	24 (18.5)
	Pharmacokinetics	14 (10.8)
	Others	6 (4.6)

* Pharm.D = Doctor of Pharmacy

† M.Phil = Masters in philosophy

‡ PhD = Doctor of philosophy

§ ICU: Intensive care unit

### Attitude towards research

Out of a maximum score i.e., 5 (100%) for the attitude towards PBR, the respondents obtained a median score of 4 (IQR = 0), demonstrating a fair positive attitude towards PBR.

Majority of the respondents (n = 117, 90%) agreed (Strongly agreed (SA) + Agreed (A)) with the statement *“Pharmacy practice research is significant in recognizing and examining complications in pharmacy”* (Median = 4, IQR = 1). Similarly, most of the respondents (n = 112, 86.2%) agreed (Strongly agreed (SA) + Agreed (A)) with Item no. 2 (Median = 4, IQR = 1). For details please refer to Table 2.

**Table 2:** Attitude towards research.

Attitude Item	Strongly disagree N (%)	Disagree N (%)	Neutral N (%)	Agree N (%)	Strongly agree N (%)	Median (IQR)
1. I like to read research studies related to pharmacy practice.	2 (1.5)	4 (3.1)	26 (20.0)	73 (56.2)	25 (19.2)	4 (0)
2. I shall be glad to be a part of research projects related to pharmacy practice.	2 (1.5)	6 (4.6)	10 (7.7)	74 (56.9)	38 (29.2)	**4 (1)**
3. I have faith in my capabilities to apprehend research and related terminologies concerned with pharmacy practice.	3 (2.3)	5 (3.8)	18 (13.8)	68 (52.3)	36 (27.7)	4 (1)
4. I am confident about my skills for designing research project related to pharmacy practice.	2 (1.5)	4 (3.1)	31 (23.8)	61 (46.9)	32 (24.6)	4 (1)
5. I am self-reliant in my skill for evaluating research terms of their application to pharmacy practice.	2 (1.5)	5 (3.8)	30 (23.1)	60 (46.2)	33 (25.4)	4 (2)
6. Pharmacy practice research is significant in recognizing and examining complications in pharmacy.	3 (2.3)	1 (0.8)	9 (6.9)	69 (53.1)	48 (36.9)	**4 (1)**
7. Pharmacy practice research is vital in pharmacy decision-making.	2 (1.5)	4 (3.1)	19 (14.6)	57 (43.8)	48 (36.9)	4 (1)
**Overall**						4 (0)

IQR = Interquartile range

***Note*:** Attitude was assessed by giving 1 to Strongly disagree, 2 to Disagree, 3 to Neutral, 4 to Agree and 5 to Strongly agree.

### Perceived importance of research in pharmacy practice

Out of a maximum score i.e., 5 (100%) for the perceptions towards PBR, the respondents obtained a median score of 4 (IQR = 0), demonstrating good perceptions towards PBR.

Majority of the respondents (n = 113, 86.9%) agreed (Strongly agreed (SA) + Agreed (A)) with the statement *“It is crucial to be well-informed of the research fitting to the practice of pharmacy”* (Median = 4, IQR = 1). Similarly, most of the respondents (n = 111, 85.4%) agreed (Strongly agreed (SA) + Agreed (A)) with Item no. 5 (Median = 4, IQR = 1). For details please refer to Table 3.

**Table 3:** Perceived importance of research in pharmacy practice.

Perception Item	Strongly disagree N (%)	Disagree N (%)	Neutral N (%)	Agree N (%)	Strongly agree N (%)	Median (IQR)
1. Research is of pivotal importance for a pharmacist.	-	6 (4.6)	28 (21.5)	51 (39.2)	45 (34.6)	4 (2)
2. It is crucial to be well-informed of the research fitting to the practice of pharmacy.	-	5 (3.8)	14 (10.8)	70 (53.8)	41 (31.5)	**4 (1)**
3. My routine practice relies on evidence based pharmacy practice research.	2 (1.5)	13 (10.0)	34 (26.2)	63 (48.5)	18 (13.8)	4 (1)
4. Being a practicing pharmacist the research findings are inapt to me.*	15 (11.5)	62 (47.7)	26 (20.0)	18 (13.8)	9 (6.9)	4 (1)
5. Research is necessary to advance patient care.	2 (1.5)	5 (3.8)	10 (7.7)	58 (44.6)	55 (42.3)	**4 (1)**
6. Research is essential for my professional recognition.	1 (0.8)	7 (5.4)	27 (20.8)	63 (48.5)	32 (24.6)	4 (1)
7. Research is of pivotal importance for my self-assurance.	-	9 (6.9)	30 (23.1)	62 (47.7)	29 (22.3)	4 (1)
**Overall**						4 (0)

IQR = Interquartile range

*Negative statement.

***Note*:** Perceptions were assessed by giving 1 to Strongly disagree, 2 to Disagree, 3 to Neutral, 4 to Agree and 5 to Strongly agree. For the question 4, it was assessed by giving 5 to Strongly disagree, 4 to Disagree, 3 to Neutral, 2 to Agree and 1 to Strongly agree.

### Willingness to participate in research

Out of a maximum score i.e., 5 (100%) for the willingness towards PBR, the respondents obtained a median score of 4 (IQR = 1), demonstrating high willingness towards PBR.

Majority of the respondents (n = 112, 86.2%) agreed (Strongly agreed (SA) + Agreed (A)) with the statement *“I have the required abilities to participate in research”* (Median = 4, IQR = 0). Similarly, most of the respondents (n = 112, 86.2%) agreed (Strongly agreed (SA) + Agreed (A)) with Item no. 4 (Median = 4, IQR = 1). For details please refer to Table 4.

**Table 4:** Willingness to participate in research.

Willingness Item	Strongly disagree N (%)	Disagree N (%)	Neutral N (%)	Agree N (%)	Strongly agree N (%)	Median (IQR)
1. There are increased chances that I would be a part of research.	3 (2.3)	**-**	28 (21.5)	50 (38.5)	24 (18.5)	4 (1)
2. I have the required abilities to participate in research.	2 (1.5)	5 (3.8)	21 (16.2)	78 (66.0)	24 (18.5)	**4 (0)**
3. I would contribute in research only in case I am paid for it. *	23 (17.7)	32 (24.6)	39 (30.0)	22 (16.9)	14 (10.8)	3 (2)
4. I would need observation to participate in research.	3 (2.3)	7 (5.4)	18 (13.8)	67 (51.5)	35 (26.9)	**4 (1)**
5.My routine activities do not allow me to indulge in research.*	2 (1.5)	24 (18.5)	26 (20.0)	50 (38.5)	28 (21.5)	4 (1)
6. I am equipped to take out time for executing research during work.	-	23 (17.7)	38 (29.2)	56 (43.1)	13 (10.0)	4 (1)
7. I shall like to take on pharmacy centered research.	1 (0.8)	5 (3.8)	28 (21.5)	71 (54.6)	25 (19.2)	4 (1)
**Overall**						4 (1)

IQR = Interquartile range

*Negative statement.

***Note*:** Willingness was assessed by giving 1 to Strongly disagree, 2 to Disagree, 3 to Neutral, 4 to Agree and 5 to Strongly agree. For the questions 3 and 5, it was assessed by giving 5 to Strongly disagree, 4 to Disagree, 3 to Neutral, 2 to Agree and 1 to Strongly agree.

### Motivators and barriers to take part in research

Out of a maximum score i.e., 5 (100%) the respondents obtained a median score of 4 (IQR = 0) for the motivation towards PBR thus demonstrating increased motivation towards PBR.

Majority of the respondents (n = 122, 93.9%) agreed (Strongly agreed (SA) + Agreed (A)) with the statement *“Provide better services and increased patient care”* (Median = 4, IQR = 1). Similarly, most of the respondents (n = 121, 93.1%) agreed (Strongly agreed (SA) + Agreed (A)) with Item no. 1 (Median = 4, IQR = 1). For details please refer to Table 5.

**Table 5:** Motivational factors to participate in research.

Motivational Item	Strongly disagree N (%)	Disagree N (%)	Neutral N (%)	Agree N (%)	Strongly agree N (%)	Median (IQR)
1. The pharmacy profession would be uplifted.	1 (0.8)	2 (1.5)	6 (4.6)	68 (52.3)	53 (40.8)	**4 (1)**
2. Provides an opportunity to offer a chance to gain knowledge related to disease management.	-	3 (2.3)	8 (6.2)	67 (51.5)	52 (40.0)	4 (1)
3. Provide better services and increased patient care.	1 (0.8)	2 (1.5)	5 (3.8)	69 (53.1)	53 (40.8)	**4 (1)**
4. Offer monetary reward.	8 (6.2)	22 (16.9)	46 (35.4)	39 (30.0)	15 (11.5)	3 (1)
5. Awareness of clinical research.	-	6 (4.6)	28 (21.5)	58 (44.6)	38 (29.2)	4 (2)
6. Backing from a colleague.	7 (5.4)	10 (7.7)	50 (38.5)	48 (36.9)	15 (11.5)	3 (1)
7. Aids me in CME (continuing medical education).	1 (0.8)	6 (4.6)	34 (26.2)	65 (50.0)	24 (8.5)	4 (1)
8. Gives me personal gratification.	1 (0.8)	8 (6.2)	28 (21.5)	62 (47.7)	31 (23.8)	4 (1)
9. Accessibility of replacement for my research time.	2 (1.5)	5 (3.8)	55 (42.3)	55 (42.3)	13 (10.0)	4 (1)
10. To sustain research activities.	3 (2.3)	4 (3.1)	24 (18.5)	66 (50.8)	33 (25.4)	4 (1)
**Overall**						4 (0)

IQR = Interquartile range

***Note*:** Attitude was assessed by giving 1 to Strongly disagree, 2 to Disagree, 3 to Neutral, 4 to Agree and 5 to Strongly agree.

A number of barriers limiting pharmacists’ participation in PBR were identified. The most common barrier reported by most of the pharmacists was lack of time (n = 31, 23.8%) followed by lack of incentives (n = 21, 16.2%), lack of support (n = 19, 14.6%) and lack of knowledge (n = 15, 11.5%). Please refer to Table 6.

**Table 6:** Barriers in practice-based research.

Barriers in research	N (%)
No personal interest	10 (7.7)
Not enough staff	8 (6.2)
Not aware of opportunity	9 (6.9)
Lack of time	31 (23.8)
Never been asked to	11 (8.5)
Lack of incentives	21 (16.2)
Lack of knowledge	15 (11.5)
Lack of support	19 (14.6)
Lack of research	6 (4.6)

### Difference in hospital pharmacists’ attitude, perceptions, willingness, and motivation towards PBR

Independent samples Mann-Whitney U Test showed a significant association (*p-value* <0.05) between gender and attitude, perceptions, and willingness of hospital pharmacists towards PBR. Independent samples Kruskal-Wallis Test showed that area of practice was found to be statistically associated (p-value = 0.031) with the motivation of hospital pharmacists (Table 7).

**Table 7:** Test of statistical significance of variation in the hospital pharmacists’ attitude, perceptions, willingness, and motivation towards PBR by their characteristics.

Variables		Attitude		*P-value*	Perception		*P-value*	Willingness		*P-value*	Motivation		*P-value*
		Negative	Positive		Poor	Good		Less	More		Low	High	
**Age**	less than 25	12 (9.2)	55 (42.3)	0.485	18 (13.8)	49 (37.7)	0.774	18 (13.8)	49 (37.7)	0.579	8 (6.2)	59 (45.4)	0.665
	25–30	5 (3.8)	35 (26.9)		7 (5.4)	33 (25.4)		11 (8.5)	29 (22.3)		6 (4.6)	34 (26.2)	
	31–35	0 (0.0)	12 (9.2)		1 (0.8)	11 (8.5)		3 (2.3)	9 (6.9)		0 (0.0)	12 (9.2)	
	36–40	1 (0.8)	4 (3.1)		1 (0.8)	4 (3.1)		2 (1.5)	3 (2.3)		0 (0.0)	5 (3.8)	
	41–45	0 (0.0)	4 (3.1)		0 (0.0)	4 (3.1)		0 (0.0)	4 (3.1)		0 (0.0)	4 (3.1)	
	46–50	1 (0.8)	1 (0.8)		1 (0.8)	1 (0.8)		0 (0.0)	2 (1.5)		0 (0.0)	2 (1.5)	
**Gender**	Male	7 (5.4)	68 (52.3)	**0.001**	9 (6.9)	66 (50.8)	**0.015**	14 (10.8)	61 (46.9)	**0.025**	3 (2.3)	72 (55.4)	0.072
	Female	12 (9.2)	43 (33.1)		19 (14.6)	36 (27.7)		20 (15.4)	35 (26.9)		11 (8.5)	44 (33.8)	
**Qualification**	Pharm. D	16 (12.3)	92 (70.8)	0.183	24 (18.5)	84 (64.7)	0.634	29 (22.3)	79 (60.8)	0.271	11 (8.5)	97 (74.6)	0.176
	M.Phil.	1 (0.8)	16 (12.3)		3 (2.3)	14 (10.8)		3 (2.3)	14 (10.8)		3 (2.3)	14 (10.8)	
	PhD	2 (1.5)	3 (2.3)		1 (0.8)	4 (3.1)		2 (1.5)	3 (2.3)		0 (0.0)	5 (3.8)	
**Years of experience in pharmacy**	< 2	12 (9.2)	55 (42.3)	0.686	19 (14.6)	48 (36.9)	0.206	19 (14.6)	48 (36.9)	0.525	10 (7.7)	57 (43.8)	0.779
	2–5	3 (2.3)	39 (30.0)		6 (4.6)	36 (27.7)		10 (7.7)	32 (24.6)		4 (3.1)	38 (29.2)	
	6–10	2 (1.5)	12 (9.2)		2 (1.5)	12 (9.2)		3 (2.3)	11 (8.5)		0 (0.0)	14 (10.8)	
	>10	2 (1.5)	5 (3.8)		1 (0.8)	6 (4.6)		2 (1.5)	5 (3.8)		0 (0.0)	7 (5.4)	
**Clinical training**	Yes	7 (5.4)	49 (37.7)	0.203	12 (9.2)	44 (33.8)	0.654	12 (9.2)	44 (33.8)	0.518	7 (5.4)	49 (37.7)	0.513
	No	12 (9.2)	62 (47.7)		16 (12.3)	58 (44.6)		22 (16.9)	52 (40.0)		7 (5.4)	67 (51.5)	
**Other board certified qualification**	Yes	4 (3.1)	18 (13.8)	0.945	5 (3.8)	17 (13.1)	0.554	4 (3.1)	18 (13.8)	0.495	2 (1.5)	20 (15.4)	0.232
	No	15 (11.5)	93 (71.5)		2 (17.7)	85 (65.4)		30 (23.1)	78 (60.0)		12 (9.2)	96 (73.8)	
**Area of practice**	Outpatient	8 (6.2)	24 (18.5)	0.491	6 (4.6)	26 (20.0)	0.412	9 (6.9)	23 (17.7)	0.529	2 (1.5)	30 (23.1)	**0.031**
	Inpatient	5 (3.8)	61 (46.9)		14 (10.8)	52 (40.0)		14 (10.8)	52 (40.0)		5 (3.8)	61 (46.9)	
	Emergency	4 (3.1)	13 (10.0)		4 (3.1)	13 (10.0)		5 (3.8)	12 (9.2)		3 (2.3)	14 (10.8)	
	Others	2 (1.5)	13 (10.0)		4 (3.1)	11 (8.5)		6 (4.6)	9 (6.9)		4 (3.1)	11 (8.5)	

Results of the logistic regression analysis revealed that hospital pharmacists practicing in the inpatient settings had 4.56 times more positive attitude towards PBR (OR = 4.56, 95%CI = 1.07─19.42, *p-value* = 0.040) as compared to those practicing in the outpatient settings. Furthermore, male hospital pharmacists had 8.86 times increased motivation towards PBR (OR = 8.86, 95%CI = 1.15–53.74, *p-value* = 0.017) as compared to female hospital pharmacists. Similarly, hospital pharmacists practicing in the outpatient settings had 23.51 times increased motivation towards PBR (OR = 23.51, 95%CI = 2.04─271.53, *p-value* = 0.011) and hospital pharmacists practicing in the inpatient settings had 12.24 times increased motivation towards PBR (OR = 12.24, 95%CI = 1.61─94.66, *p-value* = 0.016) as compared to those practicing in other settings (Table 8).

**Table 8:** Logistic regression analysis of factors associated with hospital pharmacists’ attitude, perceptions, willingness, and motivation towards PBR.

Variables		Attitude	Perception	Willingness	Motivation
		OR, 95%CI, P-value	OR, 95%CI, P-value	OR, 95%CI, P-value	OR, 95%CI, P-value
**Age**	Less than 25	Ref. (1.0)	Ref. (1.0)	0.00, 0.00, 0.99	Ref. (1.0)
	25–30	1.11, 0.24–4.02, 0.886	0.75, 0.20–2.84, 0.671	0.00, 0.00, 0.99	0.28, 0.04–2.09, 0.216
	31–35	0.00, 0.00, 0.998	1.26, 0.12–13.61, 0.852	0.00, 0.00, 0.99	0.00, 0.00, 0.99
	36–40	0.00, 0.00, 0.99	0.04, 0.00–1.71, 0.094	0.00, 0.00, 0.99	0.80, 0.00, 1.00
	41–45	0.00, 0.00, 0.99	0.00, 0.00, 0.99	0.15, 0.00, 1.00	0.00, 0.00, 0.99
	46–50	0.00, 0.00, 0.99	0.00, 0.00, 0.99	Ref. (1.0)	0.00, 0.00, 1.00
**Gender**	Male	Ref. (1.0)	Ref. (1.0)	Ref. (1.0)	8.86, 1.15–53.74, **0.017**
	Female	0.44, 1.26–1.53, 0.197	0.16, 0.05–0.52, 0.002	0.40, 0.15–1.06, 0.065	Ref. (1.0)
**Qualification**	Pharm.D	Ref. (1.0)	Ref. (1.0)	Ref. (1.0)	0.00, 0.00, 1.00
	M.Phil.	0.00, 0.00, 0.99	26.00, 0.51–132.13, 0.104	0.00, 0.00, 0.99	0.00, 0.00, 1.00
	PhD	0.00, 0.00, 0.99	0.00, 0.00, 0.99	0.00, 0.00, 0.99	Ref. (1.0)
**Years of Experience**	<2	Ref. (1.0)	Ref. (1.0)	Ref. (1.0)	0.00, 0.00, 0.99
	2–5	2.02, 0.46–8.84,0.350	1.944, 0.602–6.282, 0.266	0.16, 0.42–3.21, 0.78	0.00, 0.00, 0.99
	6–10	0.33, 0.04–3.03, 0.328	3.373, 0.307–37.048, 0.320	0.66, 0.12–3.52, 0.622	2.43, 0.00, 1.00
	>10	0.18, 0.01–4.74, 0.307	2.82, 0.10–82.10, 0.547	0.33, 0.02–7.23, 0.485	Ref. (1.0)
**Clinical Training**	Yes	Ref. (1.0)	Ref. (1.0)	Ref. (1.0)	0.26, 0.05–1.32, 0.104
	No	0.20, 0.11–1.59, 0.442	1.39, 1.49–3.92, 0.537	0.80, 0.32–2.01, 0.634	Ref. (1.0)
**Other board certified qualifications**	Yes	Ref. (1.0)	Ref. (1.0)	Ref. (1.0)	2.82, 0.40–19.98, 0.297
	No	0.98, 0.23–5.03, 1.084	0.53, 0.13–2.18, 0.376	0.68, 0.18–2.53, 0.566	Ref. (1.0)
**Area of practice**	Outpatient	Ref. (1.0)	Ref. (1.0)	Ref. (1.0)	23.51, 2.04–271.53, **0.011**
	Inpatient	4.56, 1.07–19.42, **0.040**	0.60, 0.18–2.05, 0.417	1.10, 0.34–3.40, 0.863	12.24, 1.61–94.66, **0.016**
	Emergency	1.14, 0.21–6.07, 0.877	0.87, 0.17–4.50, 0.863	0.80, 0.18–3.49, 0.761	5.33, 0.50–57.45, 0.168
	Others	2.30, 0.32–15.50, 0.391	0.45, 0.08–2.47, 0.36	0.44, 0.10–1.90, 0.274	Ref. (1.0)

## Discussion

The current study set out to determine the attitude, perception, willingness, barriers and motivation towards PBR among hospital pharmacists in Pakistan. Some studies conducted in developing countries revealed that the pharmacists were least interested in conducting research [4, 18, 19]. In contrast to this, the results of the present study showed that Pakistani hospital pharmacists were not only well aware of the importance of PBR in emerging pharmacy practice but were also willing to participate in PBR. The outcome of the survey indicated that pharmacists understood the significance and worth of research for their practice, profession, and patient care. A large number of hospital pharmacists were willing to participate in research. However, several barriers related to lack of time, support, incentives and never being asked to participate in research were also reported.

Findings of the current study revealed that hospital pharmacists had a fair positive attitude towards PBR. Participants strongly agreed/agreed that *“Pharmacy practice research is significant in recognizing and examining complications in pharmacy”* and *“Pharmacy practice research is vital in pharmacy decision-making”*. This is in line with the findings of a study conducted by Sultana et al. [3]. PBR has beneficial impact on pharmacists in terms of obtaining knowledge, improving expertise through evidence-based practice and rationalizing the decision making process in the provision of patient-oriented services. A significant role can be played by the hospital pharmacists in research due to their direct involvement in provision of optimal care to the patients. Moreover, they are in an ideal position for implementing evidence based pharmaceutical care to the patients. In the current study, 89% of the participants strongly agreed/agreed with their interest in conducting research which is comparable to the findings of a Qatari study in which 70% of pharmacists showed interest in being a part of PBR [4]. Nearly half of the respondents agreed that they understood research terminologies. This is in contrast to the previous studies that demonstrated a relatively poor knowledge of standard health related research terms among physicians and pharmacists [13, 20]. If the pharmacists are unaware about the research terminologies then they would be unable to assess any paper provided by the pharmaceutical firms or other healthcare professionals. The enhancement of critical thinking skills and familiarity with the research terminologies is of great importance for hospital pharmacists. Our study showed that the hospital pharmacists practicing in the inpatient settings had more positive attitude towards PBR as compared to those practicing in the outpatient settings. It might be due to the fact that the pharmacists working in inpatient settings had more interaction with the patients as compared to those working in outpatient settings who were merely involved in administrative tasks like dispensing of medicines. However, future studies are needed to explore the relationship between pharmacists’ attitude and hospital setting.

Respondents in the current study demonstrated good perceptions towards PBR. Many participants disagreed with the statement *“Being a practicing pharmacist the research findings are inapt to me”* justifying the importance of PBR. Majority of the pharmacists agreed with the statement *“Research is of pivotal importance for a pharmacist”*. Similar positive perceptions have been reported in an Australian study [21]. The findings of the current study revealed an increased willingness of respondents towards PBR. Participants strongly agreed/agreed that *“I have the required abilities to participate in research”* and *“I would need observation to participate in research”*. This is in contrast with the findings of a study conducted in the United Kingdom (UK) where only 32 to 50% of the pharmacists were interested in conducting research [22]. A possible explanation for such differences could be attributed to the difference in the study settings since the pharmacists working in above mentioned study were working in the community settings as opposed to current study where pharmacists were employed in hospital settings. Findings of the current study also depicted that more than half of the respondents were confident about their skills and ability to carry out a research project. This is comparable with the findings of other studies [4, 7, 17, 21]. In contrast to other studies nearly one quarter of the participants were of the opinion that monetary benefits did not have an influence on their research interests [17, 21].

Study participants also showed an increased motivation towards PBR. Participants strongly agreed/agreed with the statement *“Provide better services and increased patient care”* and *“The pharmacy profession would be uplifted”*. The factors that motivated the pharmacists to take part in PBR included the desire of uplifting the pharmacy profession, provision of better services and increased role in patient care, continuous medical education and for sustaining research activities. Similar motivational factors have been reported in other studies [13, 23]. Furthermore, male hospital pharmacists had increased motivation towards PBR as compared to female hospital pharmacists. However, the exact reason behind the relation of gender with motivation needs to be explored.

Our study revealed that the major barriers that hindered the participation of pharmacists in research were the lack of time, lack of incentives, lack of support, and lack of knowledge to conduct PBR. Lack of time has been reported to be a major barrier in various previously published studies [13, 21, 24]. In concordance with the findings of our study, other studies have also reported lack of support as one of the barriers [7, 21]. Moreover, lack of awareness and never being approached has been termed as barriers elsewhere [13]. Lacks of confidence, inadequate skills, knowledge, and training have been termed as barriers in multiple studies [7, 21, 22]. It was quite encouraging that the respondents had trust on their capabilities, knowledge and ability to carry out research. The responsibility lies on the national and international organizations to overcome the barriers faced by pharmacists having positive attitude, perception and willingness to participate in PBR.

This study has few limitations as well. First, the study was conducted in a single city of Pakistan, so results could not be generalizable to the entire country. However, the healthcare system and curriculum of healthcare professionals is similar across the country and the findings are likely to be similar for entire country. Second, some estimations of CI in Table 8 were very wide; the point estimate was not in the midpoint of the CI, which indicated a lack of precision. Data was rechecked to ensure that the CI was actually asymmetrical rather than being a calculation error. It was possibly due to the limited sample size or high variability in responses of participants. Last, the possibility of non-response bias and self-report bias could not be ruled out due to the utilization of self-administered questionnaire. There might be some differences in the accuracy or completeness of the recollections retrieved by the participants, and an under or over-reporting of attitude, perceptions, willingness, and motivation scales.

## Conclusion

The respondents had fair positive attitude, good perceptions, increased motivation and willingness towards PBR which is a promising finding. The most common barrier limiting pharmacists’ participation in PBR was lack of time followed by lack of incentives and lack of support. Male gender was found to be statistically associated with positive attitude, perceptions, and willingness towards PBR. Similarly, area of practice was found to be statistically associated with the motivation of hospital pharmacists.

## Supporting information

S1 Appendix Questionnaire of the study. (DOCX)

S1 File SPSS file. (SAV)
