# Supplementary material for: Attitude, perception, willingness, motivation and barriers to practice-based research: A cross-sectional survey of hospital pharmacists in Lahore, Punjab, Pakistan
Source: PLoS One. 2018 Sep 7;13(9):e0203568. doi: 10.1371/journal.pone.0203568 (PMC6128607; doi:10.1371/journal.pone.0203568)
Supplement: S1 Appendix — (DOCX) [file pone.0203568.s001.docx]

**Attitude, Perception, Willingness, Motivation and Barriers to Practice-Based Research: a cross-sectional survey of Hospital Pharmacists in Lahore, Punjab, Pakistan**

We are conducting a study to assess the ‘**Attitude, Perception, Willingness, Motivation and Barriers to Practice-Based Research’**. This is an anonymous questionnaire and we would like to assure, that all information provided will be utilized for the purpose of research and kept confidential. We thank you for taking the time to assist us in our educational endeavors. Your participation is voluntary and there is no penalty, if you do not participate.

| **Section 1** |
| --- |

**Q1.Have you done research before?**

Yes

No

| **Section 2** |
| --- |

**PHARMACY PRACTICE RESEARCH** is research that focuses on the practice of pharmacy and establishes a solid evidence base for new pharmacy practices and services.

PLEASE INDICATE YOUR LEVEL OF AGREEMENT WITH EACH OF THE FOLLOWING STATEMENTS RELATING TO PHARMACY PRACTICE RESEARCH BY TICKING THE APPROPRIATE BOX:

| **A) Attitude towards Research** |  |  |  |  |  |
| --- | --- | --- | --- | --- | --- |
|  | **Strongly Agree**  **5** | **Agree**  **4** | **Neutral**  **3** | **Disagree**  **2** | **Strongly**  **Disagree**  **1** |
| 1. I like to read research studies related to pharmacy practice. |  |  |  |  |  |
| 1. I shall be glad to be a part of research projects related to pharmacy practice. |  |  |  |  |  |
| 1. I have faith in my capabilities to apprehend research and related terminologies concerned with pharmacy practice. |  |  |  |  |  |
| 1. I am confident about my skills for designing research project related to pharmacy practice. |  |  |  |  |  |
| 1. I am self-reliant in my skill for evaluating research terms of their application to pharmacy practice. |  |  |  |  |  |
| 1. Pharmacy practice research is significant in recognizing and examining complications in pharmacy. |  |  |  |  |  |
| 1. Pharmacy practice research is vital in pharmacy decision-making. |  |  |  |  |  |
| **B) Perceived importance of research in pharmacy practice** | | | | | |
|  | **Strongly Agree**  **5** | **Agree**  **4** | **Neutral**  **3** | **Disagree**  **2** | **Strongly**  **Disagree**  **1** |
| 1. Research is of pivotal importance for a pharmacist. |  |  |  |  |  |
| 1. It is crucial to be well-informed of the research fitting to the practice of pharmacy. |  |  |  |  |  |
| 1. My routine practice relies on evidence based pharmacy practice research |  |  |  |  |  |
| 1. Being a practicing pharmacist the research findings are inapt to me.* |  |  |  |  |  |
| 1. Research is necessary to advance patient care. |  |  |  |  |  |
| 1. Research is essential for my professional recognition. |  |  |  |  |  |
| 1. Research is of pivotal importance for my self-assurance. |  |  |  |  |  |
| **C) Willingness to participate in research** | | | | | |
|  | **Strongly Agree**  **5** | **Agree**  **4** | **Neutral**  **3** | **Disagree**  **2** | **Strongly**  **Disagree**  **1** |
| 1. There are increased chances that I would be a part of research. |  |  |  |  |  |
| 1. I have the required abilities to participate in research. |  |  |  |  |  |
| 1. I would contribute in research only in case I am paid for it. * |  |  |  |  |  |
| 1. I would need observation to participate in research. |  |  |  |  |  |
| 1. My routine activities do not allow me to indulge in research.* |  |  |  |  |  |
| 1. I am equipped to take out time for executing research during work. |  |  |  |  |  |
| 1. I shall like to take on pharmacy centered research. |  |  |  |  |  |

| **Section 3** |
| --- |

**Q3.What factors may influence your decision to participate in a research project.**

Please indicate your level of agreement with each of the following statements relating to your participation in a research project by ticking the appropriate box.

| 1. The pharmacy profession would be uplifted. | **Strongly Agree**  **5** | **Agree**  **4** | **Neutral**  **3** | **Disagree**  **2** | **Strongly**  **Disagree**  **1** |
| --- | --- | --- | --- | --- | --- |
| 1. Provides an opportunity to offer a chance to gain knowledge related to disease management. |  |  |  |  |  |
| 1. Provide better services and increased patient care. |  |  |  |  |  |
| 1. Offer monetary reward. |  |  |  |  |  |
| 1. Awareness of clinical research. |  |  |  |  |  |
| 1. Backing from a colleague. |  |  |  |  |  |
| 1. Aids me in CME (continuing medical education). |  |  |  |  |  |
| 1. Gives me personal gratification. |  |  |  |  |  |
| 1. Accessibility of replacement for my research time. |  |  |  |  |  |
| 1. To sustain research activities. |  |  |  |  |  |

| **Section 4** |
| --- |

**Q4.What do you think are your barriers or obstacles in taking part in research? (You may tick all that apply)**

No personal interest

Not enough staff

Not aware of opportunity

Lack of time

Never been asked to

Lack of incentives

Lack of knowledge

Lack of support

Specify___________________

Lack of research

| **Section 5** |
| --- |

**Q5. Area of research that interests you? (Tick all that apply)**

Pharmacy Administration (Quality Management)

Basic Science (Pharmacogenomics, New structural drugs)

Pharmacoeconomics /Epidemiology

Pharmacy practice

Hospital Pharmacy

Therapeutics

Pharmacokinetics

Others________________

**Q6. If you have to choose a clinical study, which area interests you? (Tick all that apply)**

Pediatrics/neonates

ICU

Cardiac

Transplant

Nephrology

Surgery

Internal medicine

Geriatrics

Infectious diseases

Oncology

| **Section 6** |
| --- |

**Demographic**

**Q7. Age**

Less than 25

25-30

31-35

36-40

41-45

46-50

51 and above

**Q8.** **Gender**

Male

Female

**Q9. Qualifications acquired. (Tick all that apply)**

Pharm. D

Master

PhD

**Q10. Have you done any clinical training? (Residency or Fellowship)**

Yes

No

**Q11. Do you have any other board certified qualifications?**

Yes

No

**If yes, specify________________________**

**Q12. Current area of practice**

Outpatient setting

Inpatient setting

Emergency

Others

**Q13. Number of years of pharmacy experience in your field:**

<2 2-5

6-10 >10

**Thank you for taking time to complete this survey**
